# Supplementary material for: Hot spot prediction in protein-protein interactions by an ensemble system
Source: BMC Syst Biol. 2018 Dec 31;12(Suppl 9):132. doi: 10.1186/s12918-018-0665-8 (PMC6311905; doi:10.1186/s12918-018-0665-8)
Supplement: Supplementary file 2 — All relevant MATLAB code. A simple Matlab implement of our predictor. (ZIP 19,742 kb) [file 12918_2018_665_MOESM2_ESM.zip › Additional file 2/Read ASA/ASA website/ASEdb/1DANT.htm]

 NetSurfP - Protein Surface Accessibility and Secondary Structure Predictions

  

|  |  |  |
| --- | --- | --- |
|  |  | NetSurfP - Protein Surface Accessibility and Secondary Structure Predictions  Technical University of Denmark |

  


---

```
# For publication of results, please cite:
# A generic method for assignment of reliability scores applied to solvent accessibility predictions.
# Bent Petersen, Thomas Nordahl Petersen, Pernille Andersen, Morten Nielsen and Claus Lundegaard
# BMC Structural Biology 2009, 9:51 doi:10.1186/1472-6807-9-51
#
# Column 1: Class assignment - B for buried or E for Exposed - Threshold: 25% exposure, but not based on RSA
# Column 2: Amino acid
# Column 3: Sequence name
# Column 4: Amino acid number
# Column 5: Relative Surface Accessibility - RSA
# Column 6: Absolute Surface Accessibility
# Column 7: Z-fit score for RSA prediction
# Column 8: Probability for Alpha-Helix
# Column 9: Probability for Beta-strand
# Column 10: Probability for Coil
E N  1DANT                  1    0.916 134.088   0.090   0.003   0.003   0.994
E T  1DANT                  2    0.441  61.208  -0.471   0.020   0.205   0.775
E V  1DANT                  3    0.348  53.488  -0.770   0.021   0.451   0.528
E A  1DANT                  4    0.475  52.312   1.070   0.023   0.655   0.322
B A  1DANT                  5    0.216  23.748  -1.184   0.021   0.756   0.223
B Y  1DANT                  6    0.268  57.272   0.631   0.018   0.846   0.136
E N  1DANT                  7    0.429  62.732  -0.637   0.003   0.718   0.279
B L  1DANT                  8    0.116  21.276   0.460   0.003   0.718   0.279
E T  1DANT                  9    0.299  41.430   0.313   0.018   0.846   0.136
B W  1DANT                 10    0.126  30.303  -0.148   0.001   0.900   0.099
E K  1DANT                 11    0.383  78.845   0.507   0.018   0.846   0.136
B S  1DANT                 12    0.193  22.596  -1.527   0.023   0.655   0.322
E T  1DANT                 13    0.346  48.046  -0.670   0.005   0.336   0.660
E N  1DANT                 14    0.535  78.368  -0.222   0.004   0.138   0.858
B F  1DANT                 15    0.160  32.192  -0.477   0.020   0.205   0.775
E K  1DANT                 16    0.408  84.008   0.568   0.004   0.616   0.381
B T  1DANT                 17    0.194  26.977   0.325   0.002   0.816   0.182
B I  1DANT                 18    0.104  19.259   0.461   0.011   0.918   0.071
B L  1DANT                 19    0.102  18.749   0.426   0.001   0.900   0.099
E E  1DANT                 20    0.369  64.447   0.070   0.001   0.900   0.099
B W  1DANT                 21    0.103  24.868  -0.067   0.002   0.816   0.182
E E  1DANT                 22    0.427  74.632   0.218   0.004   0.514   0.481
E P  1DANT                 23    0.328  46.557  -0.954   0.005   0.336   0.660
E K  1DANT                 24    0.538 110.687   0.408   0.005   0.336   0.660
E P  1DANT                 25    0.296  42.017  -1.260   0.004   0.138   0.858
E V  1DANT                 26    0.391  60.035  -0.803   0.004   0.085   0.910
E N  1DANT                 27    0.640  93.681  -1.277   0.004   0.085   0.910
B Q  1DANT                 28    0.235  41.882   0.534   0.004   0.420   0.576
E V  1DANT                 29    0.269  41.330  -0.393   0.002   0.816   0.182
B Y  1DANT                 30    0.116  24.746   0.879   0.001   0.900   0.099
B T  1DANT                 31    0.137  19.057   0.431   0.001   0.959   0.040
B V  1DANT                 32    0.045   6.855   0.814   0.001   0.959   0.040
B Q  1DANT                 33    0.265  47.311   0.624   0.001   0.959   0.040
B I  1DANT                 34    0.078  14.356   0.064   0.001   0.900   0.099
B S  1DANT                 35    0.110  12.869  -0.462   0.023   0.655   0.322
E T  1DANT                 36    0.473  65.550  -0.080   0.021   0.451   0.528
E K  1DANT                 37    0.504 103.735   0.126   0.056   0.142   0.802
E S  1DANT                 38    0.484  56.666  -1.816   0.053   0.043   0.903
E G  1DANT                 39    0.657  51.737   0.055   0.053   0.043   0.903
E D  1DANT                 40    0.554  79.846   0.002   0.052   0.084   0.864
B W  1DANT                 41    0.239  57.600   0.583   0.125   0.227   0.648
E K  1DANT                 42    0.524 107.807   0.646   0.135   0.317   0.548
E S  1DANT                 43    0.420  49.247   0.518   0.125   0.227   0.648
E K  1DANT                 44    0.294  60.435   1.347   0.135   0.317   0.548
B C  1DANT                 45    0.056   7.876   0.149   0.079   0.592   0.329
B F  1DANT                 46    0.149  29.904  -0.012   0.085   0.775   0.140
E Y  1DANT                 47    0.336  71.739   0.305   0.087   0.683   0.230
B T  1DANT                 48    0.146  20.306  -0.235   0.074   0.484   0.442
E T  1DANT                 49    0.392  54.315  -0.552   0.064   0.216   0.721
E D  1DANT                 50    0.555  80.019  -0.979   0.113   0.087   0.800
B T  1DANT                 51    0.274  37.976  -0.466   0.191   0.086   0.723
E E  1DANT                 52    0.419  73.234  -0.351   0.307   0.165   0.527
B C  1DANT                 53    0.080  11.260   0.115   0.191   0.086   0.723
E D  1DANT                 54    0.409  58.894   0.092   0.278   0.093   0.628
B L  1DANT                 55    0.133  24.389   0.380   0.354   0.048   0.598
B T  1DANT                 56    0.247  34.287   0.122   0.622   0.015   0.363
E D  1DANT                 57    0.523  75.379  -0.520   0.879   0.010   0.111
E E  1DANT                 58    0.522  91.281   0.479   0.879   0.010   0.111
B I  1DANT                 59    0.074  13.746  -0.493   0.879   0.010   0.111
B V  1DANT                 60    0.230  35.351   0.630   0.802   0.014   0.185
E K  1DANT                 61    0.608 125.045   1.313   0.802   0.014   0.185
E D  1DANT                 62    0.401  57.799   0.150   0.694   0.003   0.303
B V  1DANT                 63    0.056   8.638   0.258   0.694   0.003   0.303
E K  1DANT                 64    0.421  86.600   0.709   0.717   0.014   0.269
E Q  1DANT                 65    0.520  92.926   0.171   0.751   0.050   0.199
E T  1DANT                 66    0.280  38.836   0.526   0.701   0.107   0.192
B Y  1DANT                 67    0.092  19.703   0.920   0.578   0.229   0.194
B L  1DANT                 68    0.236  43.267  -0.046   0.487   0.370   0.144
B A  1DANT                 69    0.118  13.037  -0.521   0.487   0.370   0.144
E R  1DANT                 70    0.290  66.456   0.631   0.487   0.370   0.144
B V  1DANT                 71    0.062   9.483   0.226   0.373   0.401   0.226
B F  1DANT                 72    0.139  27.958   0.177   0.373   0.401   0.226
B S  1DANT                 73    0.267  31.257  -0.822   0.352   0.332   0.316
E Y  1DANT                 74    0.298  63.683   0.528   0.321   0.252   0.427
E P  1DANT                 75    0.333  47.182  -0.758   0.191   0.086   0.723
E A  1DANT                 76    0.324  35.705  -0.168   0.181   0.016   0.803
E G  1DANT                 77    0.634  49.919  -0.669   0.181   0.016   0.803
E N  1DANT                 78    0.667  97.605  -0.738   0.115   0.016   0.868
E V  1DANT                 79    0.304  46.771  -1.309   0.115   0.016   0.868
E E  1DANT                 80    0.906 158.313   0.631   0.003   0.003   0.994

Explain the output.  Go back.
```

---
